# Starvation Genocide and the Triumph of Raphael Lemkin

**DOI:** 10.5041/RMMJ.10466

**Published:** 2022-04-26

**Authors:** George M. Weisz

**Affiliations:** 1 School of Humanities, University of New South Wales, Sydney, Australia; 2 School of Humanities, University of New England, Armidale, Australia

**Keywords:** Genocide, Lemkin, starvation

## Abstract

Today, in the 21st century, most people are aware of the term genocide. However, few people are aware that this term only entered the English language in the 1940s, as a result of the dedicated work of a brilliant and successful man who deprived himself of a private family life so that he could be free to fight for his ideas. Although Raphael Lemkin was instrumental in the recognition of genocide by the United Nations, he died too early and was buried with no honor. This paper reviews the life and work of Raphael Lemkin, and his triumph in seeing genocide recognized as a crime.

## RAPHAEL LEMKIN’S IMPORTANCE IN THE DEFINING OF GENOCIDE

Raphael Lemkin was born into a Jewish family on June 24, 1900 in the village of Bezwodne, near today’s Byelorussian town of Vaulkovisk, just east of the Polish border. His father was a farmer; however, his mother was an intellectual. Home schooled by his mother, Lemkin was a voracious reader, and from a young age deeply impressed and horrified by the atrocities of man on man throughout history.^1^ Lemkin studied both philology and law at the University of Lwów (now Lviv, Ukraine). His love of learning led to additional studies in universities in Heidelberg, Paris, and Rome. He eventually learned a total of 12 languages, but settled on the law, graduating with a doctorate from Lwów.^1^,^2^

Shortly after graduating with his doctorate in law, Lemkin was appointed Deputy Prosecutor in Warsaw. In 1933, having been prohibited from leaving Warsaw, Lemkin gave a presentation by proxy at a League of Nations conference in Madrid on international law; it focused on the prevention and punishment of any “destruction of collective cultural heritage” (or “Vandalism”), as well as “destruction of people” (or “Barbarism”).^3^,^4^

His theory, although not adopted in Madrid, was further developed into a protection from the destruction of national, religious, or ethnic life. Lemkin’s world view was well established by the time the Nazis came to power. As a result, leaving behind his very large family, Lemkin escaped to Sweden via the Baltics in 1939.

The parting words of Lemkin’s mother were prophetic:

You realize, Raphael, that it is you, not we, who needs protection now … of all of us only you do not live the life of love. You are the lonely and the loveless one. Still, you have been carrying the burden of your idea, which is based on love … We know you will continue your work, for the protection of peoples. Unfortunately, it is needed now more than ever before.^2^^(p58)^

**Figure 1:**
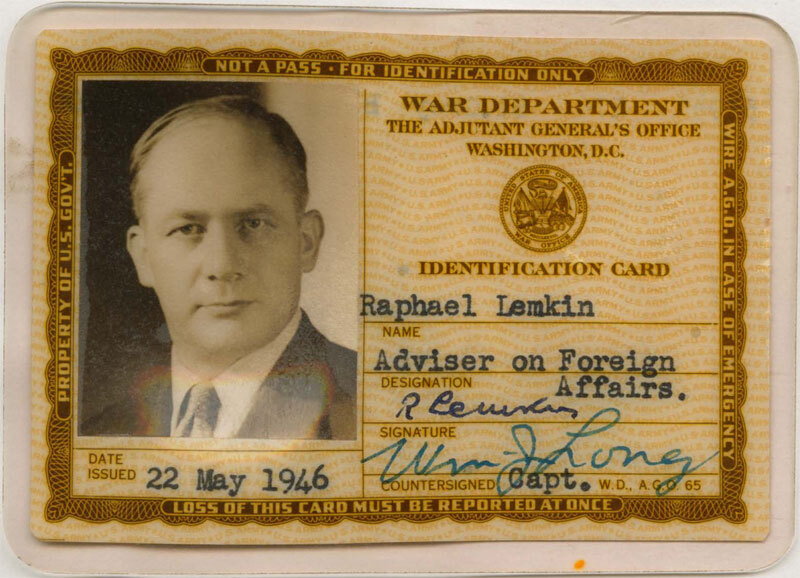
Photo of Raphael Lemkin on his War Department Identification Card. Photo by usafdo from Find a Grave (https://www.findagrave.com/memorial/25927143/raphael-lemkin).

In Stockholm, Lemkin taught Law and added further to his language skills. Within a year, he was offered teaching positions at Yale and Duke universities. After a long journey through Asia and Japan, he reached North America in 1941. At the end of World War II, he was employed by the US War Department as an instructor and interpreter at the Nuremberg Trials, assisting the Chief Prosecutor. His US War Department Identity Card reveals Lemkin’s probing eyes and serious look (Figure 1).

### Creation of the Term Genocide

Lemkin noted that starvation was a weapon of subjugation and/or extermination—a renewal of an old atrocity cited by history books, now being replayed in his own time. Such atrocity, as described in 1941 by Winston Churchill, was “a crime without a name.” Lemkin brought together the Greek word, *genos* (nation, race, or tribe), and *cide* from the Latin word for killing, to create a new term, *genocide*, an incredibly appropriate term for the atrocities he had observed.^4^

The burden of his observations led Lemkin to obsessively push hard for official recognition of and sanctions against genocide. He was considered unpleasant company in the corridors of the United Nations (UN) and was often socially ignored. In 1942, upon learning about the executions in Poland, Lemkin wrote to President Roosevelt and asked him to take action, but received the disappointing reply, “be patient.”^2^ Lemkin remained an internationalist in his fight against racism and for the codification of genocide, while some 49 of his family members perished, in part from hunger in the forests of Byelorussia, with the rest perishing in Treblinka, their “grave in the clouds above” (to paraphrase Paul Celan^5^). Lemkin remained an unbiased theoretician and sought to elevate starvation to the status of a crime against humanity.^2^–^4^

### Developing a Code of Genocide Under Nazi Rule

In 1944 Lemkin published his influential book, *Axis Rule in Occupied Europe*,^3^ followed by numerous other books, articles, and conference presentations, all of which pushed for the codification and recognition of genocide. In his 1944 book, Lemkin fleshed out the definition of genocide as “a coordinated plan of different actions aiming at the destruction of essential foundations of the life of national groups, with the aim of annihilating the groups themselves.”^3^^(p79)^ Lemkin defined several aspects of genocide, as implemented by the Nazis. Essentially, genocide could be either by overt killing or any other covert means affecting health, nourishment, family life, and care of children.^3^,^4^,^6^–^10^ These aspects could be summarized as follows:^2^

1. *Political:* The occupation of a country, separating it into easily controlled zones, and establishing puppet states. These areas were to be Germanized.
2. *Social:* Intellectuals and clergy were to be removed (erased), to undermine and destroy the social structure, making the population easier to control, as was done in Poland, Slovenia, and to thousands in the Netherlands.
3. *Cultural:* Everything relying on a non-German local language (courts, schools, literature, or journalistic publications) was to be Germanized. Furthermore, the German cultural heritage was considered essential for maintaining the life of the group, and any insult to or destruction of Germanic objects was judged as vandalism.
4. *Moral:* Corruption of children’s education was effectively performed via pornographic teaching and the promotion of alcoholism.
5. *Religious:* The clergy were to be undermined by rearranging church teachings and organization, as enforcing the nomination of Nazi-approved patriarchs.
6. *Biological:* There was an active effort to reduce non-Germanic births by promoting abortions and undernourishment, thereby increasing infant and child mortality.
7. *Physical:* The extermination of sub-humans (by shooting, carbon dioxide, or cyanide gas), defined as Jews, Sinti, or any other person not considered able to contribute to Germanic society (mentally retarded, mentally ill, homosexuals, etc.)
8. *Economic:* Racially based food allocations, expropriation of monies or items of monetary value, and property confiscations. This effectively reduced standards of living; the so-called “sub-humans” had no protection from cold in the winter, and were unable to purchase food or other necessities. The only exceptions were for relatives to German people, who had to undergo full re-education, namely Germanization.

### Lemkin’s Triumph: United Nations Recognition of Genocide as a Crime

Although genocide entered the record of indictments at the Nuremberg Trials, it was not part of the final judgement against the Nazi leaders, this despite the passionate pleas of Lemkin. Although Lemkin was not a registered member of the tribunal, his influence on one the chief prosecutors, Justice Jackson, was obvious. Jackson quoted Lemkin’s theory, but did not relate to it as a legal principle. While all four prosecutors agreed with the principles of indictment at the Nuremburg Trials, they placed different emphases on them.^11^ Both British prosecutor Sir Shawcross and French prosecutor August Champetier de Ribes unequivocally stated that a main tool of the Nazis was starvation.^11^

Lemkin’s triumph was finally realized when the UN General Assembly affirmed that genocide was a crime under international law, whether the crime was committed on religious, racial, political, or any other grounds. Adopted in December 1948 under the title of the Convention on the Prevention and Punishment of the Crime of Genocide, this definition would gradually be ratified by member states. Genocide crimes were defined as:

any of the following acts committed with intent to destroy, in whole or in part, a national, ethnical, racial or religious group, as such:

- Killing members of the group;
- Causing serious bodily or mental harm to members of the group;
- Deliberately inflicting on the group conditions of life calculated to bring about its physical destruction in whole or in part;
- Imposing measures intended to prevent births within the group;
- Forcibly transferring children of the group to another group.^12^

### Final Years

**Figure 2:**
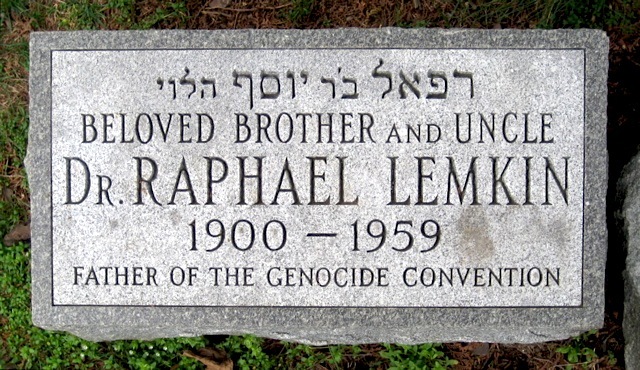
Grave of Raphael Lemkin. Photo by Ronzoni at Find a Grave (https://www.findagrave.com/memorial/25927143/raphael-lemkin).

Lemkin would be the recipient of numerous awards and was nominated for the Nobel Peace Prize ten times, including one nomination by Winston Churchill. Despite his fame and accomplishments, Lemkin died at the age of 59, a penniless member of the New York Community, and was buried in the Mount Hebron Cemetery, Flushing, Queens County, NY (Figure 2). Only seven people attended his funeral, three people short of the ten necessary to conduct the burial prayers in accordance with his Jewish ancestry.

## DISCUSSION: STARVATION AS GENOCIDE?

The concept of Starvation Genocide has been of great interest to this author, who as a young child witnessed varying aspects of the phenomenon.

Lemkin was a visionary in his battle to formalize the criminalization of starvation. All who were aware of the issues clearly understood his definition, which called for recognition of starvation as a deliberate tool for genocide, and therefore a crime.^9^–^11^,^13^–^19^

While the recognition of genocide by the UN was an important first step, forced labor, starvation, or imposing conditions leading to the diseases upon a given population were not directly defined as genocide. In fact, it was only in 2018 that the UN officially adopted a resolution that specifically recognized starvation and food insecurity, among other acts, as illegal acts of warfare.^20^ Nevertheless, by association, starvation can clearly be recognized as a type of genocide.

It has now been proven that despite the contributions of climactic catastrophes, most, if not all, famines worldwide have been due not to the lack of food, but rather to the lack of availability, poor distribution, and political interventions.^6^–^10^ Socio-economists have additionally shown that there was indeed sufficient food to feed the entire global population in the world of the twentieth century.^6^–^10^ Starvation is indeed the shame of the century as described by the economist/sociologist Steven Devereux, who discusses how 70 million people starved to death despite sufficient food being available to feed the entire global population.^6^

### Starvation Genocide as an Act of War

Simplistically speaking, starvation and famine are two different things: famine is the extreme scarcity of food whereas starvation is the result of that scarcity. It is therefore intriguing that famine can be graded by severity of impact, as measured by mortality. Howe and Devereux proposed five grades of famine ranging from minor to catastrophic, i.e. less than 1,000 deaths and at least 1,000,000 or more, respectively.^7^ While the causes of famine are complex, several researchers have clearly shown that the end result, starvation, has been used throughout history as an act of warfare.^6^,^16^–^19^,^21^

It is not an understatement to say that the twentieth century began with starvation being used as an act of war. During World War I, the Allied Navy developed a war strategy that involved the severing of food supplies to the Middle East (today’s Lebanon, Israel, Syria) via the Mediterranean, while the Ottoman Empire additionally imposed food requisitions and simultaneously cut off the land route from the East. These blockades exacerbated a current locust invasion in the region, adding a climacteric impact to the starvation resulting from the blockades. Those combined actions resulted in 200,000 deaths.^16^

During 1932–33, the lack of cooperation by Ukrainian farmers with Soviet collectivization led to food confiscation. The event, termed as Holodomor, resulted in close to 4 million Ukrainian deaths and was paralleled by the Soviet Famine with 10 million deaths.^16^

### The Nazis: Starvation Genocide as an Official Policy

Nothing was technically and militaristically more conducive to starvation than the new Nazi Order in Europe. Lemkin quoted Marshal von Rundstedt at the Wehrmacht Academy in 1943:

One of the great mistakes of 1918 was to spare the civil life of the enemy countries. … We are therefore obliged to destroy at least a third of their inhabitants. The only means is organized underfeeding which in this case is better than machine guns.^4^^(p79)^

A 4-year program was officially promoted by Marshal Goering in 1941 and referred to as the “Green Plan” for food preservation. He is recorded to have said: “This continual concern for aliens must come to an end once and for all … I could not care less when you say that people under your administration are dying of hunger. Let them perish so long as no German starves.”^22^^(p253)^ Goering had based his statement on the Fuehrer’s thesis (Table talks that ranged from 1941 to 1944^23^): “I don’t see why a German who eats a piece of bread should torment himself with the idea that the soil that produced this bread has been won by the sword.”^24^^(p844)^

In parallel with the Green Plan, the secretary of the Food Ministry, Herbert Backe, was tasked to develop a *Weisung* (directive rather than a Law) that would be referred to as the “Hunger Plan.”^25^–^30^ Designed to assure food supplies for the Wehrmacht (Army) in the third year of the war to come, it was initially introduced on May 2, 1941, and included two main points: 31

The War can only be continued, if the entire Wehrmacht is fed from Russia in the third year of war.

If we take what we need out of the country, there can be no doubt that many millions of people will die of starvation.^31^

In accord with this plan, the loss of human life was expected to reach 20–30 million lives. Under Backe’s direction, an effective scheme for supervision of food production was introduced. For example, punitive measures against delinquent peasants became severe: the punishment for withholding their crops was imprisonment instead of financial fines.^28^

The deliberate starvation against non-submitting populations during World War II has been well described. The starvation resulting from these plans alone led to millions of deaths—clearly exceeding the above-mentioned definition of a catastrophic famine. While the severe winter of 1941 clearly added to the impact of this humanly imposed famine, the actual number of deaths from starvation are far more than could have been achieved by the weather alone. Having been deliberately implemented, this “plan” can clearly be defined as starvation genocide. Within the first year of the Nazi invasion of Russia, 3 million Soviet prisoners died. Between 1939 and 1944, more than 5 million Russians/Ukrainians had died, and these numbers do not include the starvation-related deaths in the rest of occupied Europe, the concentration camps, and beyond.^16^,^17^ Holland’s *Hongerwinter*, which began in November 1944, was imposed by German occupying forces as a reaction to Dutch resistance efforts. The German army instituted a food embargo over Western Netherlands. By April 1945, there was a food availability of a mere 400 calories/day/person, and 18,000 deaths were recorded.^16^

How do these horrendous historical events and figures fit into Lemkin’s definition of genocide? As discussed above, Lemkin was clear on the fact that genocide could be covert as well as overt.

Historically, the Nazi regime was not the first to employ hunger as a weapon, but theirs was certainly the most sophisticated methodology. According to Lemkin, a combined Genocide Code (killing and starvation) would represent the highest degree of criminality. This was clearly described by Commandant Rudolf Hoess, who testified at the Nuremberg trials stated that in Auschwitz, apart from the 2.5 million “exterminated by gassing or burning”^32^^para414^ in Auschwitz, another 500,000 died from starvation and disease.^32^

Hence, Lemkin’s demands that starvation be considered another category of genocide is not unreasonable. Whether committed by covert or overt means, with or without intent, deliberate starvation constitutes a conspiracy to commit a crime against humanity.

## LEMKIN’S TRIUMPH—HUMANITY’S FAILURE

Lemkin’s initial definition of genocide has undergone numerous additional interpretations. The UN was the first to officially recognize starvation, but, as discussed above, their definition had certain limitations. Researchers, historians, philosophers, and sociologists in the post-war decades have detailed their interpretations of genocide, some extending while other restricting the definition.^21^,^33^–^36^ Since starvation was not overtly included as part of the definition of genocide, some scholars have stepped out to place more emphasis on that category.

Despite differences, genocide researchers all agree with Lemkin that the intent to destroy, be it for cultural, linguistic, political, religious, or economic reasons, is an integral aspect of genocide.^4^,^35^

Unlike the UN, well-known sociologist and historian Nusan Porter, from a Jewish family, did not hesitate to include starvation as a platform for genocide. His definition appears to have been derived from both experience and theory. Born in Rovno, Ukraine in December 1944, his family eventually emigrated to the United States. The combination of personal, theoretical, and objective training helped him develop a definition of genocide that is more objective than theoretical by pointing out the deliberateness of the destruction by the perpetrators, whether achieved by means of starvation or economic or biologic means.^37^,^38^

The UN’s 1948 adoption of the Convention on the Prevention and Punishment of the Crime of Genocide was intended to prevent further genocides.^3^ However, there have been more genocides listed in the second half of the twentieth century compared to the first half. The atrocities observed since World War II, particularly related to starvation, have moved from Russia, to Asia, to Africa, as detailed by Howe and Devereux; hence, it is worthwhile to take a closer look at their 2004 data.^7^^(p6)^ While climacteric conditions clearly have impacted the severity of major and catastrophic famines, armed conflict and governmental policies have played a key role in the majority of famine-related deaths since World War II.^7^^(p6)^

It is clear to this author that while death is inevitable in armed conflict, if food scarcity or famine are deliberate strategies to subdue another, the only fit term for this is starvation genocide.

A simple Google search for “Raphael Lemkin” gives more than 200,000 hits–reflecting his relative worldwide recognition today. It is tragic that Lemkin’s true fame came only after his death. His efforts have given an all-inclusive name to deliberate starvation and other crimes committed against a targeted population: genocide. However, despite the official recognition of genocide as a crime by the UN, and of starvation as a war crime by other international bodies, humanity has failed to eradicate starvation genocide in the twentieth century, and is now failing in this current century. Is it too late for us to bring an end to starvation genocide?

## Abbreviations

UN: United Nations
